# A Translational Framework of Educational Neuroscience in Learning Disorders

**DOI:** 10.3389/fnint.2018.00025

**Published:** 2018-07-04

**Authors:** Thomas Dresler, Stephanie Bugden, Camilo Gouet, Marie Lallier, Darlene G. Oliveira, Pedro Pinheiro-Chagas, Ana C. Pires, Yunqi Wang, Camila Zugarramurdi, Janaina Weissheimer

**Affiliations:** ^1^LEAD Graduate School & Research Network, University of Tübingen, Tübingen, Germany; ^2^Department of Psychiatry and Psychotherapy, University of Tübingen, Tübingen, Germany; ^3^Department of Psychology, University of Pennsylvania, Philadelphia, PA, United States; ^4^The Numerical Cognition Lab, Department of Psychology, Brain and Mind Institute, University of Western Ontario, London, ON, Canada; ^5^Laboratorio de Neurociencias Cognitivas, Escuela de Psicología, Pontificia Universidad Católica de Chile, Santiago, Chile; ^6^Basque Center on Cognition, Brain and Language, San Sebastián, Spain; ^7^Instituto Presbiteriano Mackenzie, Universidade Presbiteriana Mackenzie, São Paulo, Brazil; ^8^Cognitive Neuroimaging Unit, Institut National de la Santé et de la Recherche Médicale, Paris, France; ^9^Laboratory of Behavioral and Cognitive Neuroscience, Stanford Human Intracranial Cognitive Electrophysiology Program, Department of Neurology and Neurological Sciences, Stanford University, Stanford, CA, United States; ^10^Centro de Investigación Básica en Psicología, Facultad de Psicología, Universidad de la República, Montevideo, Uruguay; ^11^School of International Studies, Zhejiang University, Hangzhou, China; ^12^Brain Institute, Federal University of Rio Grande do Norte, Natal, Brazil

**Keywords:** neuroimaging, learning disorders, dyscalculia, dyslexia, education, translational framework

## Abstract

Neuroimaging has undergone enormous progress during the last two and a half decades. The combination of neuroscientific methods and educational practice has become a focus of interdisciplinary research in order to answer more applied questions. In this realm, conditions that hamper learning success and have deleterious effects in the population – such as learning disorders (LD) – could especially profit from neuroimaging findings. At the moment, however, there is an ongoing debate about how far neuroscientific research can go to inform the practical work in educational settings. Here, we put forward a theoretical translational framework as a method of conducting neuroimaging and bridging it to education, with a main focus on dyscalculia and dyslexia. Our work seeks to represent a theoretical but mainly empirical guide on the benefits of neuroimaging, which can help people working with different aspects of LD, who need to act collaboratively to reach the full potential of neuroimaging. We provide possible ideas regarding how neuroimaging can inform LD at different levels within our multidirectional framework, i.e., mechanisms, diagnosis/prognosis, training/intervention, and community/education. In addition, we discuss methodological, conceptual, and structural limitations that need to be addressed by future research.

## Introduction

Educational neuroscience (EN), a discipline situated between neuroscientific and psychological research, has remained rather distant from educational research (Bruer, 2016). One often claimed problem is its scarce practical application to real-world education, leaving neuroscientists virtually incapable of helping educators (Bowers, 2016). Cognitive science has been proposed as the necessary bridge from neuroscience to education (Bruer, 1997), a perspective that has produced some promising educationally oriented findings (Sigman et al., 2014). Especially in the case of learning disorders (LD; ICD-10 F81 specific developmental disorders of scholastic skills), which generally present a steady course, lack of remission, and a strong relation to the biological maturation of the brain (World Health Organization, 1992), the potential of EN to elucidate the core cognitive and neural deficits and the efficacy of training and intervention programs seems apparent (Gabrieli, 2016).

There have been some attempts to create models which try to portray the translational research process, from the basic neuroscience field to the applied educational field. One of these models (Gabrieli, 2016) proposes a pipeline organization of educational neuroscience, in which EN combines with behavioral science to motivate experimental interventions. If effective, they can be scaled to widespread classroom practice. In such model, the consideration of educational needs inspire basic research directions to prioritize development of interventions.

**FIGURE 1:**
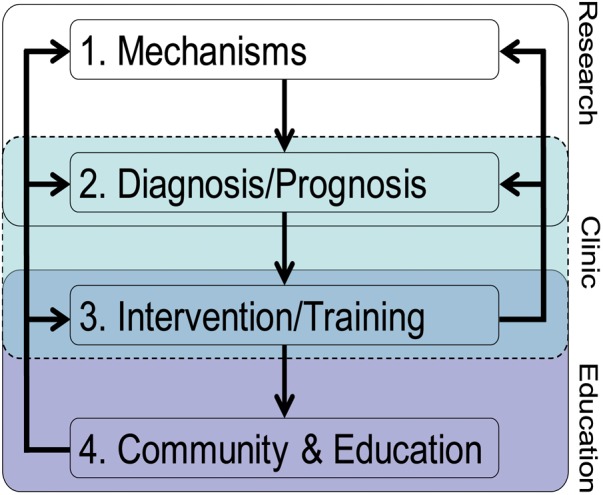
The theoretical translational framework on how EN can inform LD.

In the same line, here we put forward a theoretical translational framework for bridging EN research to education (**Figure 1**). In a way, we advance Gabrieli (2016) model by providing a perspective from which the understanding of two specific LD, dyslexia and dyscalculia, could substantially benefit. We propose that interdisciplinary collaboration across the fields of neuroscience, psychology/cognitive science, and education is essential for clarifying root causes, improving prognosis, and yielding effective remediation. These involve well-trained basic, clinical and educational researchers and practitioners (i.e., teachers and educators), which represents an additional challenge. Although our framework consists of distinct components, it is not unidirectional, allowing information to flow between the different components in either direction, making adaptations possible.

### Component 1: Mechanisms

Mechanisms represent the neural and cognitive substrates underlying a specific (dys)function (e.g., numerical processing, reading). These mechanisms should always be investigated based on multifactorial theoretical causal frameworks of LD. These theoretically based mechanisms can encompass levels of description going from macroscopic (e.g., cortical network) to microscopic scales (e.g., cellular level, even genetic level), as well as relate to brain architecture (structure), neural activity (function), or structural and functional network patterns (connectivity).

### Component 2: Diagnosis/Prognosis

Knowledge of the nature of the mechanisms involved in a specific learning (e.g., numerical processing, reading) is essential in contributing to the diagnosis and early detection (i.e., before an official diagnosis can be made) of the disorder. We suggest that a specific diagnosis is likely to benefit from the evaluation of the combination of several mechanisms, including behavioral and brain patterns (e.g., pattern/multivariate classification), even risk genes. Reliable diagnoses based on multifactorial causal frameworks of LD are likely to increase the reliability of individuals’ LD prognosis and predict accurately their developmental trajectory.

### Component 3: Intervention/Training

With a better prognostic knowledge based on a theoretically grounded diagnosis, neuroscientifically informed (often wrongly referred to as brain-based) Intervention/Training should be initiated, especially when prognosis is not advantageous.

### Component 4: Community and Education

The previous three components still lack direct applicable educational relevance, and do not offer practical solutions *per se*. To make the most of relevant findings on the previous levels, systematic knowledge transfer into the community and educational practice is necessary (Sigman et al., 2014). Prior to this, it is important to correct general and LD-related biases, neuromyths and misperceptions about the brain (Illes et al., 2010; Howard-Jones, 2014), and to question so-called brain-based programs lacking a scientific basis (Goswami, 2006).

**Table 1** gives an overview of the involved parties and relevant publications for each component.

**Table 1:** Overview of the involved parties and exemplary publications for each of the components.

Component	Involved parties	Exemplary publications
Mechanisms	Biologists, psychologists, neuroscientists, geneticists, …	Kucian et al., 2014; Lallier et al., 2017, …
Diagnosis/Prognosis	Biologists, psychologists, neuroscientists, physicians, psychometricians, …	Blomert, 2011; Hoeft et al., 2011; Dinkel et al., 2013, …
Intervention/Training	Pediatricians, psychologists, speech therapists, educators, teachers, …	Iuculano and Cohen Kadosh, 2014; Horowitz-Kraus et al., 2015, …
Community & Education	Educators, teachers, family, pediatricians, psychologists, speech therapists, science journalists, politicians/policy makers, …	Howard-Jones, 2014; Fernandez-Duque et al., 2015, …

So far, the components are mostly treated as disconnected units, which may stem from different areas of expertise (i.e., methodology, biology, neuroscience, cognitive science, clinical and social psychology, and education). For example, whereas Component 1 is mostly rooted in basic fundamental research, Component 4 focuses on applied research. In our view, one major aim of future research goes beyond the development of research on each of the components: It is about time to invest effort on how to improve the connections and interactions between the different components. EN should take a particularly important role in this endeavor. In particular, EN should focus on strengthening the links and interactions involving the community and educational cores, reflecting communication strategies between basic science and practical application. While some authors doubt the contributions of brain research to education (Bowers, 2016), in light of the promising and novel evidence that EN is providing about brain differences and how these can be translated into individualized education, we agree with Gabrieli (2016) that judicious prioritization of research directions can make those contributions substantial.

Several review and perspective articles have been published on the very topic of EN, providing welcoming, but also critical views (e.g., Goswami, 2006; Sigman et al., 2014; Bowers, 2016). As of 2017, one problem EN still faces is that its publications represent a “… meta-scientific literature, more about the promise and pitfalls of applying neuroscience to education than it is about applications of neuroscience to education” (Bruer, 2016, p. 1), which may barely reflect the “cons” of the highly interdisciplinary nature characterizing EN. In our view, studies on dyslexia and dyscalculia offer a special opportunity to support our proposed EN framework, as research conducted on these LD (i) tackles various issues addressed by all our framework components, (ii) is highly educationally relevant, and (iii) reflects a somehow chaotic pool of heterogeneous findings begging for more conclusive data. We believe that our proposed EN framework should contribute to reduce discrepant results that currently govern the LD fields.

In what follows, we provide a series of selected findings on both dyscalculia and dyslexia in order to support our theoretical EN translational approach. Each component of our framework will be addressed separately, but possible links between the components are also going to be highlighted.

## Understanding Learning Disorders Within the Framework

Dyscalculia is a LD affecting the acquisition of basic arithmetic skills, not explainable by poor schooling, deprivation, or low intelligence. It still remains unclear what underlying core deficits contribute to the inability in learning basic arithmetic (cf. Fias et al., 2013; Berteletti et al., 2014; Bugden and Ansari, 2015).

Dyslexia is a LD characterized by persistent difficulty in reading, i.e., slow and inaccurate word recognition, despite normal intelligence, schooling, and motivation. Different hypotheses have been suggested regarding underlying factors, ranging from phonological (e.g., poor/less accessible phonological representations) to sensory deficits (e.g., visual processing/visual attentional span) (cf. Goswami, 2000; Ramus and Szenkovits, 2008; Blomert, 2011; Lallier and Valdois, 2012).

### Mechanisms

**FIGURE 2:**
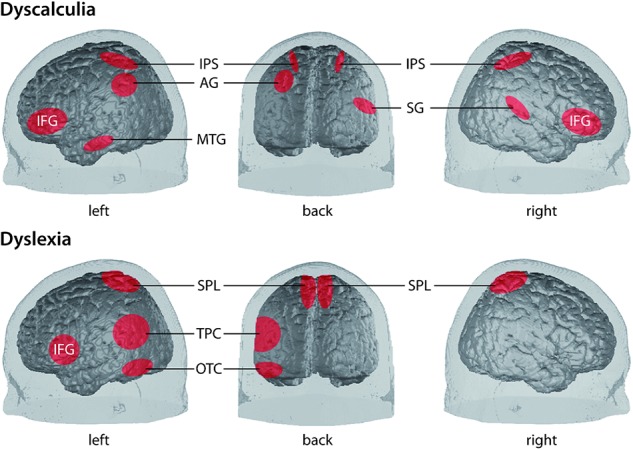
Simplified illustration of the location of involved cortical areas in the two LD dyscalculia [upper panel, from Bugden and Ansari (2015)] and dyslexia [lower panel, according to Richlan et al. (2009); Raschle et al. (2011) and Lallier and Carreiras (2017)]. AG, angular gyrus; IFG, inferior frontal gyrus; IPS, intraparietal sulcus; MTG, middle temporal gyrus; OTC, occipito-temporal cortex (comprising lateral extrastriate, fusiform, and inferior temporal regions); SG, supramarginal gyrus; SPL, superior parietal lobe; TPC, temporo-parietal cortex [comprising posterior superior temporal gyrus (STG), inferior parietal lobe].

A plethora of neurobiological studies focuses on investigating the nature of the basic causal mechanisms of these the two LD of interest here. Knowledge about these mechanisms represents an essential prerequisite to understand the processes involved in the subsequent components of our framework. Here, we summarize neuroscientific findings which we consider theoretically relevant in order to provide the reader with a short overview of the state of the art. A simplified illustration of the involved cortical areas can be found in **Figure 2**.

#### Dyscalculia

Studies on individuals with dyscalculia show some alterations in the structure, function, and connectivity, affecting mainly the parietal lobe, but also temporal and prefrontal brain regions. These findings contribute to depicting dyscalculia as a complex syndrome arising from multiple neural causes (Ashkenazi et al., 2013). Importantly, neuroimaging techniques and studies (see below) help to disentangle between the hypotheses as to whether dyscalculia is a domain-specific or domain-general phenomenon. The former hypothesis suggests that dyscalculia originates from a core deficit in processing quantity (e.g., Butterworth et al., 2011), number sense (e.g., Wilson and Dehaene, 2007), magnitude representation (e.g., Ashkenazi et al., 2009), or processing Arabic numerals (e.g., De Smedt and Gilmore, 2011). The latter hypothesis suggests that dyscalculia is associated with cognitive impairments such as verbal capacities, attention, or working memory (e.g., Berteletti et al., 2014). A third possibility could include a combination of both, contributing to multiple subtypes (e.g., Fias et al., 2013).

Structural studies found reduced gray matter volume in dyscalculia in the superior parietal lobe, including the intraparietal sulcus (IPS), but also in the fusiform gyrus (FG), lingual gyrus, (para)hippocampus, and prefrontal structures (Rotzer et al., 2008; Rykhlevskaia et al., 2009; Cappelletti and Price, 2014). The IPS is a key structure involved in the processing of numerical magnitude, which indicates a deficit in core numerical representations (Price and Ansari, 2013) and may serve as a potential target for intervention. The other structures may contribute to learning numerical processing skills by affecting memory and fact retrieval, but also attention, working memory, and visuospatial memory (Rotzer et al., 2008; Rykhlevskaia et al., 2009).

Functional studies using magnitude comparison tasks show that IPS activity is modulated by numerical distance in typically developing (TD) children, but not in dyscalculic children (e.g., Mussolin et al., 2010; Heine et al., 2013). Similar results indicating less IPS modulation in children with dyscalculia stem from studies on calculation or number line tasks (e.g., Kucian et al., 2006; Berteletti et al., 2014). Cohen Kadosh et al. (2007) showed that shortly disrupting right IPS activity using neurostimulation in healthy participants negatively impacted performance in magnitude comparison, mimicking dyscalculia. Moreover, studies showing increased activation in specific regions (supplementary motor area and prefrontal regions) in dyscalculia may indicate allocation of higher working memory and attentional resources to compensate for less fluent magnitude manipulation or difficulties in the task-related response selection, task-switching, or inhibitory processes (Kucian et al., 2011b; Cappelletti and Price, 2014).

In connectivity analyses, deficient projections in the inferior longitudinal fasciculus (ILF) and the superior longitudinal fasciculus (SLF) have been found (Rykhlevskaia et al., 2009; Kucian et al., 2014). Indicators of SLF white matter integrity correlated positively with numerical abilities in dyscalculic and TD children, highlighting the relevance of SLF integrity for numerical processing. Hence, connections of parietal magnitude representation areas with areas necessary for number processing and domain-general functions (e.g., Kucian et al., 2006; Ashkenazi et al., 2012; Cappelletti and Price, 2014) seem impaired in dyscalculia.

Altogether, these findings indicate that one prominent mechanism underlying dyscalculia is altered parietal brain activation, particularly in the right IPS (Ashkenazi et al., 2013). These findings argue for a core magnitude representation deficit. However, recent evidence suggests a role of temporal language areas (Berteletti et al., 2014) indicating impaired verbal-dependent processes which may also contribute to comorbid conditions of dyscalculia and dyslexia. Prefrontal alterations in dyscalculia suggest that domain-general functions may constitute another potential cause of this LD (Kucian et al., 2006; Ashkenazi et al., 2012).

#### Dyslexia

Neuroimaging has led dyslexia research to a better understanding of the mechanisms involved in its different subtypes, providing measures that correlate with a myriad of behavioral data (e.g., Blau et al., 2010; Boets et al., 2013). Studies mainly focus on the reading-related neural systems in children with dyslexia, and could reveal cerebral disruptions at an early age (Shaywitz et al., 2002; Blau et al., 2010). For example, Blau et al. (2010) found that in dyslexic children neural letter-speech sound integration was impaired as compared to fluently reading children. Meta-analyses (e.g., Richlan et al., 2009, 2011) indicate lower activation of the left temporo-parietal cortex (TPC), occipito-temporal cortex (OTC), and mixed findings for the inferior frontal gyrus (IFG) in dyslexia. Another recent meta-analysis by the same group of researchers concluded that there seems to be a biological unity of dyslexia, involving especially the left OTC, including the visual word-form area, with additional orthography-specific abnormalities (Martin et al., 2016).

Results also indicate possible multiple dysfunctional circuits arising from a core structural white matter deficit (Klingberg et al., 2000; Cui et al., 2016) leading to the hypothesis of a disconnection syndrome (Ashkenazi et al., 2013). The role of other subcortical and cerebellar regions is still controversial (Ashkenazi et al., 2013). Recently, Horowitz-Kraus et al. (2016) found children with reading disability (RD) to perform worse in narrative comprehension while simultaneously engaging brain regions related to executive functions and higher functional connectivity, indicating potential compensatory mechanisms.

A large number of studies have investigated the phonological deficit hypothesis (i.e., a deficit to build up the phonological representations that will be used to convert the graphemes into their corresponding phonemes), and have sought to reveal underlying neural mechanisms, emphasizing the importance of Broca’s area (note that different phonological processes may be involved in dyslexic children and adults; e.g., Georgiewa et al., 2002; Cao et al., 2006). Blomert (2011) showed that a specific ortographic-phonological binding deficit (i.e., impaired letter-speech sound integration) seems to be a proximal cause for reading deficits in dyslexia and also explain the notorious lack of reading fluency. Using multi-voxel pattern analysis and connectivity analysis (through correlational designs) Boets et al. (2013) concluded that the core deficit in dyslexia was a deficient access to phonetic representations that were otherwise intact. However, thanks to the unique temporal properties of magnetoencephalography (MEG), Molinaro et al. (2016) were able to show an impaired directional connectivity in dyslexia, going from the right auditory cortex to Broca’s area, refuting the idea of an impaired phonological access in dyslexia, but supporting a poor phonological perceptual mechanism that would cause phonological manipulation difficulties.

Interestingly, the above-mentioned MEG study (Molinaro et al., 2016) focused on neural oscillations. Indeed, it has been recently suggested that atypical neural activity in both the auditory and visual modalities could explain why some individuals suffer from dyslexia (Lallier et al., 2017). For example, according to the temporal sampling framework (Goswami, 2011) poor phonological representations in dyslexia could be explained by imprecise synchronization of oscillatory brain activity at low frequencies to the amplitude envelope of the speech signal (see also Hamalainen et al., 2012; Lizarazu et al., 2015; Molinaro et al., 2016). It remains to be seen if the promising studies on cortical oscillatory processes (Goswami, 2016) will help to better understand the mechanisms and their diagnostic/prognostic and therapeutic value.

In addition to providing fine description of a dysfunction in dyslexia, neuroimaging techniques can also help at confirming the existence of independent subtypes described at the cognitive level (Bosse et al., 2007). Based on a double-dissociation hypothesis between phonological and visual attention span disorders (deficit for processing several visual elements simultaneously in a multi-element array) in dyslexic individuals (Valdois et al., 2003), Peyrin et al. (2012) investigated whether a similar double dissociation could be revealed at the neurobiological level. They found brain activation patterns mirroring the cognitive disorders’ dissociation (Broca’s area for the phonological subtype and superior parietal lobes for the visual attention span subtype), thereby providing evidence for an association between independent brain dysfunctions and independent cognitive deficits.

As for imaging-genetic studies, a few attempts to the early identification of children with a familiar risk to develop dyslexia have been made (Mascheretti et al., 2018), resulting in the characterization of dyslexia-susceptibility genes and the molecular etiological pathways underlying the development of RD in order to better inform well-timed prevention and remediation strategies [for a recent review also see Mascheretti et al. (2017)].

### Diagnosis/Prognosis

#### Dyscalculia

Diagnosis is based on the criteria established by ICD-10 and DSM IV as a discrepancy between performance in standardized mathematics tests and normal intelligence; measured by specific screenings available. Differential diagnosis is crucial: as dyscalculia may represent a core deficit, mathematical learning disability (MLD) corresponds to low mathematical skill performance without reference to intelligence, and includes multiple deficits (Kaufmann and von Aster, 2012). Disturbances outside the core parietal regions in dyscalculia indicate different phenotypes and neuroimaging could help to theoretically and conceptually distinguish these and to evaluate different theories. Here, by revealing different underlying mechanisms (Component 1), neuroimaging could assist diagnostic methods in distinguishing specific subtypes that are generally difficult to define on mere behavioral observations.

Dumontheil and Klingberg (2012) found left IPS activation to be more sensitive to correctly classifying children as poor arithmetical performers than when only behavioral measures were considered. This shows that neuroimaging can provide additional diagnostic information without replacing behavioral data, but by increasing the validity of the existing clinical behavioral indexes. However, Dinkel et al. (2013) discuss the diagnostic potential of reliable single case fMRI data, based on the finding that altered neural patterns were found in the absence of deficits on the behavioral performance of individuals with dyscalculia. In dyscalculia, a shift of activation from the primary to higher visual systems was detected; in addition, associated fronto-parietal activation was suggested to represent a stable compensatory neural mechanism (Dinkel et al., 2013). Future connectivity analysis approaches might add complementary information to improve diagnoses.

Although prognosis is not fully determined, early identification is crucial given the high prevalence (5%) and comorbidity of dyscalculia (e.g., Rapin, 2016). Since even preverbal infants display a parietal specialization for numbers (Hyde et al., 2010), early neuroscientifically informed detection and following intervention programs are – although rather utopian at the moment – not totally implausible in the future. Without adequate intervention, dyscalculia tends to persist into adulthood and to be economically costly (Butterworth et al., 2011; Kaufmann and von Aster, 2012) which calls for more work in order to define valid prognosis criteria from an early stage.

#### Dyslexia

According to the International Dyslexia Association (IDA), the diagnosis of dyslexia involves a myriad of measures, considering dyslexia more as a multi-dimensional rather than a categorical disorder. Studies suggest that neural alterations may be present at birth or develop in early childhood prior to reading onset (Lyytinen et al., 2003). It has been demonstrated that gray matter alterations are already observable in pre-readers with a family history of dyslexia and correlate with pre-reading skills (Raschle et al., 2011). Guttorm et al. (2010) showed that ERPs in combination with familial risk status reports can be useful for early detection of children at risk, facilitating early interventions before reading problems arise. Maurer et al. (2011) followed dyslexic and TD children from 2nd to 5th grade showing that deficits contributing to dyslexia are plastic and change during development and skill acquisition, opening several windows of opportunity for effective intervention.

To identify brain mechanisms that may be critical for reading improvement in dyslexia 2.5 years after diagnosis, Hoeft et al. (2011) had participants perform a printed-word rhyme judgment task during fMRI recording to elicit the phonological analysis of orthographic input that is thought to be a core deficit. Results revealed that variations in the activity and structure of the right prefrontal brain regions predicted long-term reading improvement.

Even if the last study suggests that neuroimaging technique can inform prognosis in dyslexia, a future challenge will consist in assessing children right after diagnosis and years later longitudinally, and comparing neural and behavioral data between the two time points in order to determine how the severity of dyslexia symptoms at diagnosis onset predicts their evolution. Early identification is important because in young children the brain is potentially more malleable for the rerouting of neural circuits (Shaywitz et al., 2003). However, the effects of intervention programs should be reflected in a positive reorganization of neural networks, no matter when the remediation is initiated (Goswami, 2006).

### Training/Intervention

#### Dyscalculia

The “Diagnosis/Prognosis” component of our framework has the inherent goal of allowing to detect individuals at risk of LD and to select the appropriate intervention; hence, neuroscientifically informed interventions could be developed to directly tackle the neural mechanisms highlighted. So far, the existing computerized training tools mostly improve number comparison, but they hardly generalize to other relevant number skills (Rasanen et al., 2009). The first set of data available on behavioral and neural activation change after a number-line training, reports improved spatial representation of numbers and of arithmetic problem solving (Kucian et al., 2011a). Kucian et al. (2011a) also report an associated neural modulation in the IPS, although the timing of the measurement of these changes seems to be essential to characterize the origin of the neural modulations (synaptic vs. systemic consolidation). It has been recently found that intensive 1:1 math tutoring focused on strengthening conceptual and procedural knowledge normalizes aberrant functional brain responses in children with MLD; astonishingly, these children could not be distinguished from TD children anymore after an intervention using brain activation pattern classification (Iuculano et al., 2015).

There are some studies that try to directly transfer knowledge from our mechanistic knowledge of LD to the selection of intervention programs: Iuculano and Cohen Kadosh (2014) applied transcranial direct current stimulation (tDCS) to the posterior parietal cortex (PPC) of two dyscalculic adults while learning numerical meaning of arbitrary symbols. One participant received left cathodal (inhibitory) and right anodal (excitatory) stimulation, and the other received the opposite pattern of stimulation. Only the latter (right cathodal and left anodal) improved learning. Interestingly, healthy subjects seem to respond better to the opposite pattern (Cohen Kadosh et al., 2010). First, such findings indicate that dyscalculic and TD subjects respond to tDCS (Schroeder et al., 2017). Second they suggest that neurostimulation protocols may be one future option for remediation of dyscalculic symptoms. However, before such intervention becomes possible, response differences between dyscalculia and TD groups and between different patients need to be further clarified as dyscalculia appears rather heterogeneous at the individual level (Dinkel et al., 2013).

#### Dyslexia

Studies on training and intervention in dyslexic individuals generally focus on behavioral remediation approaches, with the goal to restore brain activation closer to that seen in normal-reading children (Temple et al., 2003). Koyama et al. (2013) employed resting-state fMRI comparing intrinsic functional connectivity among dyslexic groups receiving partial, full, or no remediation at all. Remediation groups exhibited stronger connectivity between left FG and right middle occipital gyrus, suggesting compensatory strategies changes associated with remediation, rather than cortical normalization. Valdois et al. (2014) studied a French-Spanish bilingual dyslexic girl with a severe visual attention span deficit resulting in a reduction of reading speed in both languages, but preserved phonological skills. After an intensive visual attention span intervention program, text reading improved in the two languages. In addition, comparison of pre- and post-training fMRI revealed significant activation increases in the superior parietal lobes bilaterally. The authors argue that a specific visual attention span intervention not only modulates reading performance, but further results in increased brain activity within areas known to housing visual attention span abilities. Lastly, Horowitz-Kraus et al. (2015) found that, after applying the Reading Acceleration Program, training-related increases in resting-state connectivity between specific components were positively correlated with increased word reading and reading comprehension, respectively.

Neuroimaging can visualize and quantify the neural correlates of the multiple processes that intervene between stimulus and response. At the moment, the above mentioned studies represent a good starting point for using neuroimaging to potentially inform training or intervention. In the future, it will be necessary to focus on these processes providing information that goes beyond established behavioral measurements (see Bowers, 2016). Prediction is also important to help identify which students would respond to a certain intervention and which would not. As Gabrieli (2016) puts it, we often wait too long for prolonged failure in a child’s reading achievement to initiate intervention. As the author further claims, better learning and teaching would occur if important student characteristics could be identified in the outset so that curriculum could be individualized rather than implemented on a trial-and-error basis.

### Community and Education

Despite all the knowledge gathered on the previous three components proposed here, knowledge transfer into the community, educational and clinical practice does not occur naturally, and often does not take place at all. In fact, we underline that this component of the framework is particularly neglected in EN, which represents a major problem. Besides the fact that educational systems with their curricula, quality of teacher training and applied instructions are largely determined by policy-makers in federal and state governments – which sometimes neglect available empirical scientific evidence (e.g., Communication of S. Dehaene in the French newspaper Le Monde, 20 December 2013) – there are also other problems to face. We are confident that neuroscientists and educators can tackle the following issues when working together:

(1) *Neuroscientists need to convey information appropriately to practitioners before any application can take place*. Neuromyths still prevail (Howard-Jones, 2014) and brain imaging increases credibility (Fernandez-Duque et al., 2015), which gives researchers inadvertent persuasive power. Especially, in the context of development and learning, findings tend to be transmitted overly optimistic (van Atteveldt et al., 2014). As a result, non-experts can be easily fooled by neuroscientific explanations (e.g., Weisberg et al., 2008; van Atteveldt et al., 2014), while many brain-based learning programs still lack a scientific basis (Goswami, 2006). Contrariwise, brain images can be used for a more transparent communication, and illustrate findings when speaking to teachers, policy-makers, or community (Illes et al., 2010). An additional problem is the belief that problems in education should be resolved if we know how to boost a specific brain function. However, when the media coverage largely addresses brain optimization (O’Connor et al., 2012), limitations of neuroimaging in children populations are widely underestimated (Bishop, 2013). In the same line, exaggeration in scientific news strongly correlates with exaggerated misleading news in academic press releases (Sumner et al., 2014) undermining the importance of reporting accurate rather than “sexy” results. Beyond the emotional distress that LD generates for children and parents, these above-mentioned factors hold the danger of deficient communication. Therefore, scientists should be aware of the social consequences that may be generated by neuroscientific news in the media (O’Connor et al., 2012), and essentially focus on transmitting transparent information about what conclusion is strongly supported by empirical data and – equally important – what is yet to be discovered. Specific recommendations for researchers and science communicators can, for instance, be found in van Atteveldt et al. (2014).
(2) *Practical applications based on neuroscientific knowledge should be continuously evaluated during real-world implementation to determine their effects, but also to detect missing effects or even side-effects*. Here, neuroscientists can indeed learn a great deal from educators (Bowers, 2016). EN is often dominated by neuroscientists, resulting in largely one-sided perspective and influence. Feedback and mutual information exchange are essential, as real-world educational experiences may largely differ from artificial laboratory settings. Beyond, evaluated findings always have to be weighed against the questionable validity of many currently applied practices, as educational decisions often occur without empirical scientific evidence, which could be used as guidance (Gabrieli et al., 2015).
(3) *Regarding LD, neuroscientific findings can further help to establish the fact that such disorders are indeed neurobiological and changeable conditions* which is important to resolve still widespread assumptions that performance deficits are due to the learner’s laziness, stubbornness, or lack of intelligence. Findings from intervention studies positively show that deficits and neural dysfunction in LD can be improved with training and change over time, indicating behavioral and neural plasticity. Although the myth of non-change (“Learning problems associated with developmental differences in brain function cannot be remediated by education”) is less common than others (Howard-Jones, 2014), any educational practice supporting such a myth will be particularly detrimental for children with LD. That is why it is still important to stress the potential of validated and scientifically informed interventions.

To overcome these issues, more interdisciplinary collaboration between neuroscience and real-world education is needed, which may result in new concepts and messages that are both supported by science and educationally informative (Howard-Jones, 2014). Here, mutual exchange and further cooperation between the disciplines – and the community – should be of uppermost importance for the future. Last but not less importantly, not only should scientists know about the importance of translating their work for the public, but they need to have the tools and the know-how to accomplish this important goal (Illes et al., 2010).

## General Discussion and Conclusion

In order to conclude, this section focuses on three important issues, namely, (1) what has been accomplished on the realm of neuroscience and education until this moment, (2) what are the necessary steps still to be taken, and, finally, (3) what is the added value of neuroscience to the study of LD.

Regarding the first issue, enormous progress of neuroscientific research since the 1990s resulted in important empirical data on LD. More specifically, publications on mechanisms (Component 1) have substantially increased our knowledge of the neural basis of such disabilities. For example, it has been shown that dyscalculia is neither a domain-specific nor a domain-general phenomenon, but involves both aspects. This may apply more or less to specific cases and could be used for neuroscientifically informed subtypes. From behavioral studies alone, we cannot totally tease apart these conflicting theories and how they contribute to LD. Yet with new imaging tools, such as multivariate pattern analyses (e.g., Hoeft et al., 2011; Dinkel et al., 2013; Iuculano et al., 2015), we can decode different neural signals underlying different representations and examine differences across groups. This will contribute to a better understanding of LD subgroups and hence improve differential diagnosis with potentially more neuroscientifically informed prognosis (Component 2). It will further influence the next components, as we have demonstrated with the classification approaches being applied and neural-based interventions (Component 3). The direct influence of our basic knowledge of mechanisms on educational practice (Component 4) is largely negligible so far. However, the exchange of findings between the more basic and the more applied component of our framework may represent a way to reduce neuromyths, which can influence educational practice. Knowing that the brain is plastic, develops throughout life and responds to external influence, may help educators to see LD as malleable and as conditions that can be improved, thereby increasing educators’ perceived self-efficacy.

In return, the success of specific educational techniques can influence basic research questions on underlying mechanisms (link from Component 4 to Component 1). For example, for educators it might be totally irrelevant to know why starting school later has advantages and why naps help learning (Bowers, 2016); however, from a multi-component scientific perspective, these findings result in new non-trivial knowledge which may – or may not – be helpful in guiding further research questions.

As for the second issue – the necessary steps to be taken – much less is known about the neuroscientific contributions on the mechanisms involved in LD to Diagnosis/Prognosis (Component 2) and Training/Intervention (Component 3), which is largely due to the complexity of performing longitudinal studies (e.g., costs, maintenance, dropouts). Nevertheless, such studies are strongly needed to elucidate long-term consequences and to reevaluate putative prognostic markers – especially as LD are rooted in a biological origin. Neuroprediction is a relatively recent scientific endeavor. Generally, there is evidence that prediction by using neurobiological markers represents a fruitful approach, but only when we move forward from post-analyses toward prediction and from correlational toward individual prediction; such predictive analyses are needed for translating correlational observations into educational and clinical practice (Gabrieli et al., 2015).

Early investigation may offer one successful tool. There are EEG studies even in newborns that have predictive power in revealing risk for later problems in language and reading (e.g., Guttorm et al., 2010). If simple measurements can help to detect disturbances at a preverbal stage, they could be introduced, so that children at risk could be followed and subsequent diagnostic and intervention procedures be administered. In addition, there are studies suggesting that neural markers have the potential to outperform behavioral measures (e.g., Hoeft et al., 2011) and to identify children that would benefit most from a training program (Supekar et al., 2013). Although we do not deny the problems associated with neural markers (Bowers, 2016), we consider the approach to combine behavioral and neurobiological correlates as the most promising one, as it associates the strengths inherent to each approach. To this end, study designs and analyses need to be further improved and advanced (Gabrieli et al., 2015). Component 2 (Diagnosis/Prognosis) can inform Component 3 (Training/Intervention), resulting in the choice of appropriate intervention. In return, effective or non-effective intervention on behavioral and neural levels helps to reconsider the diagnosis.

**FIGURE 3:**
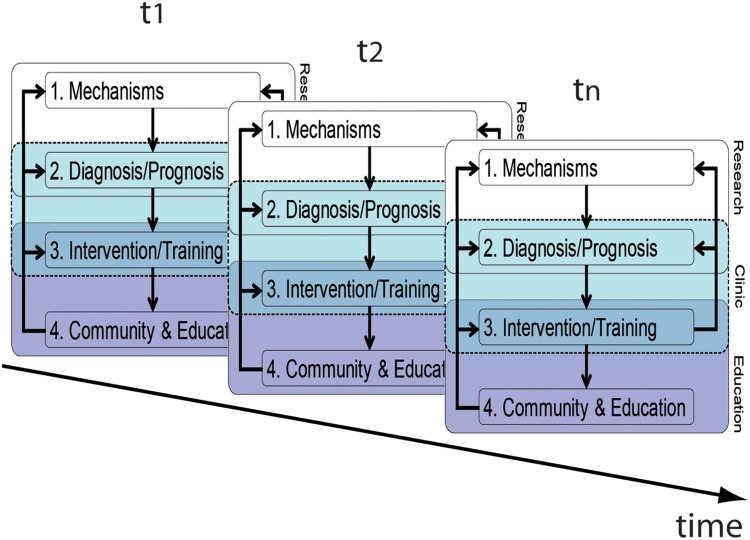
Longitudinal perspective of the translational framework. Please note that arrows connecting the components between different time-points have been omitted for clarity. Foci of the framework will change across time/age according to the respective requirements.

One necessary step is to generally take a longitudinal perspective as depicted in **Figure 3**. Only this allows us to getting a better understanding of the prognostic value. Here, we would like to emphasis that the components and associations of our framework should be considered malleable across time, which is due to individual biological changes, but also to more social, cultural, and technological influences. Our framework dependencies in early childhood education will be different from adult education, as will be the respective stakeholders; in the same manner, dependencies will be different between now and then.

Finally, regarding our third important issue, there is continuing debate on the added value of neuroscience to inform education or intervention. This debate questions whether (1) a bridge between neuroscience and education is indeed possible (Bruer, 1997; Sigman et al., 2014), (2) a gulf is to be crossed (Goswami, 2006), (3) or the idea that neuroscientists cannot help educators at all should persist (e.g., Bowers, 2016). Some possible bridging elements have been proposed in the last decade and also alluded to in our article. For example, Bishop (2013) discusses smart drugs, neurofeedback, and neurostimulation, as these directly approach core neural systems with the aim to increase learning. Yet, such studies are still in their infancy for any valid conclusion to be drawn. The question if such effects are accompanied by changes in the brain are completely irrelevant for practitioners (Bowers, 2016). We have to keep this in mind when we conduct studies: “By all means, let’s do such studies, but let’s do them because we want to find out more about the brain, and not pretend it has educational relevance” (Bishop, 2014). Such an argument might go too far, but it addresses an important issue: namely, to think more about the rationale, objectives and consequences of EN, and to cautiously consider promises made. The thought ‘from brain scan to lesson plan’ (Howard-Jones, 2011) may be appealing, but it is – until incalculable time – far away from reality.

Returning to our framework, the manifold feedback loops provide great opportunities to improve research and adapt processes, henceforth increasing knowledge and practical consequences. As an educational neuroscientist, one often faces the criticism that the only aim of their research is to put people into scanners, wire them with electrodes or apply neurostimulation (see practical issues below). However, in our view, this has never been the case. Besides, such a perspective is short-sided and may conceal and undermine the possible tremendous benefits that educational and clinical cores can get from neuroscientific findings. For example, in dyslexia, several causal hypotheses have been proposed, which to some extent can be distinguished and supported by neuroimaging. The neuroscientific discovery of independent dyslexic subtypes (e.g., Peyrin et al., 2012) can feed back to improving our knowledge of the underlying mechanisms, which in turn helps to improve and develop (non-neural) diagnostic procedures, respectively. Neuroscientifically informed procedures can improve diagnostic reliability by resulting in innovative behavioral assessments, and be applied without using any neuroscientific technique in the future. To further illustrate this point, our knowledge of the neural effects of sleep or short-term naps on the cellular level (Sigman et al., 2014) provides the scientific basis for the findings related to the benefits of naps on declarative memory in children (Lemos et al., 2014). This may in turn result in policy changes regarding the potential of a delayed school start or the introduction of nap breaks within the school routine (see also Bowers, 2016).

We would like to stress that we are aware of the many limitations (methodological, conceptual, and structural) that still exist to bridge the gap between neuroscience and education, and of the dangers that emerge from an inconsiderate trust in neuroscience, especially when LD is considered (see section “Community and Education”). For example, (1) there are practical issues that are inherent to most neuroimaging techniques, such as the fact that it is not feasible to scan each child before entering school or even the big group of kids with familial history of LD. As the practical limitation is especially inherent to fMRI, it more or less also applies to the other imaging techniques. (2) Moreover, there are unsolved ethical issues that are related to preventive diagnostics/early detection, such as the negative impact of labeling (i.e., danger of stigmatizing), miscommunication about the meaning of a risk (e.g., Gigerenzer et al., 2007), or dealing with implications of type one and type two error.

Methods need to be further developed and, in particular, problems that appear when children have to be tested and evaluated with heavy neuroscientific protocols need to be fully taken into account. In addition, most studies have been run with small samples which limit the reliability and validity of the available findings to an unknown extent. Even though neuroscience can inform education and clinical practice, we are aware that it will never replace the use of psychometric tests, neuropsychological testing normed across a large number of participants or diagnostic decision-making processes involving multiple concerned parties (e.g., educators, family, pediatricians, psychologists, and speech therapists). Even if reliable findings appear at the group level, single subject variability is a major draw-back for making possible conclusions with a high degree of confidence applying to all individuals (Dinkel et al., 2013). This problem, of course, prevents from formulating a solid prognosis or diagnosis. Besides, ethical and societal issues need to be considered (such as the cost and the local availability of the protocols used) if neural markers are clearly demonstrated as solid ways to enhance the quality of prediction of typical and atypical developmental individual trajectories (Gabrieli et al., 2015).

All in all, we argue that neuroimaging is in general potentially helpful to reveal the underlying cognitive mechanisms associated with LD, thereby improving the precision of the differential diagnoses. Furthermore, it can help to detect children at risk early on, to better understand the prognosis, and to develop more effective interventions. Finally, neuroimaging can – when addressed adequately – be used as a powerful illustrative tool to improve the communication of scientific results to educators, policy-makers and the community in general. While some authors doubt the contributions of brain research to education, we strongly agree with Gabrieli et al. (2015) when they argue, in light of the promising and novel evidence that EN is providing about brain differences and how these can be translated into individualized education, that the predictive power of neuroscientific studies expresses both a practical and humanitarian possibility for improving individuals’ lives.

## Author Contributions

TD, SB, CG, PP-C, and AP drafted the part on dyscalculia. ML, DO, CZ, YW, and JW drafted the part on dyslexia. All authors contributed to the integration of the respective parts into the translational framework. TD and JW finalized the manuscript. All authors have approved of the final version of this manuscript. All authors contributed to the conception of the manuscript.

## Conflict of Interest Statement

The authors declare that the research was conducted in the absence of any commercial or financial relationships that could be construed as a potential conflict of interest. The reviewer MIN and handling Editor declared their shared affiliation.
